# A scoping review on bio-aerosols in healthcare and the dental environment

**DOI:** 10.1371/journal.pone.0178007

**Published:** 2017-05-22

**Authors:** Charifa Zemouri, Hans de Soet, Wim Crielaard, Alexa Laheij

**Affiliations:** Department of Preventive Dentistry, Academic Centre for Dentistry Amsterdam, University of Amsterdam & Vrije Universiteit Amsterdam, Amsterdam, The Netherlands; Beijing Institute of Microbiology and Epidemiology, CHINA

## Abstract

**Background:**

Bio-aerosols originate from different sources and their potentially pathogenic nature may form a hazard to healthcare workers and patients. So far no extensive review on existing evidence regarding bio-aerosols is available.

**Objectives:**

This study aimed to review evidence on bio-aerosols in healthcare and the dental setting. The objectives were 1) What are the sources that generate bio-aerosols?; 2) What is the microbial load and composition of bio-aerosols and how were they measured?; and 3) What is the hazard posed by pathogenic micro-organisms transported via the aerosol route of transmission?

**Methods:**

Systematic scoping review design. Searched in PubMed and EMBASE from inception to 09-03-2016. References were screened and selected based on abstract and full text according to eligibility criteria. Full text articles were assessed for inclusion and summarized. The results are presented in three separate objectives and summarized for an overview of evidence.

**Results:**

The search yielded 5,823 studies, of which 62 were included. Dental hand pieces were found to generate aerosols in the dental settings. Another 30 sources from human activities, interventions and daily cleaning performances in the hospital also generate aerosols. Fifty-five bacterial species, 45 fungi genera and ten viruses were identified in a hospital setting and 16 bacterial and 23 fungal species in the dental environment. Patients with certain risk factors had a higher chance to acquire *Legionella* in hospitals. Such infections can lead to irreversible septic shock and death. Only a few studies found that bio-aerosol generating procedures resulted in transmission of infectious diseases or allergic reactions.

**Conclusion:**

Bio-aerosols are generated via multiple sources such as different interventions, instruments and human activity. Bio-aerosols compositions reported are heterogeneous in their microbiological composition dependent on the setting and methodology. *Legionella* species were found to be a bio-aerosol dependent hazard to elderly and patients with respiratory complaints. But all aerosols can be can be hazardous to both patients and healthcare workers.

## Introduction

Aerosols are defined as liquid or solid particles suspended in the air by humans, animals, instruments, or machines. Bio-aerosols are aerosols consisting of particles of any kind of organism [1, 2]. The characteristics of bio-aerosols differ depending on environmental influences such as humidity, air flow, and temperature. Aerosols, which are responsible for the transmission of airborne micro-organisms by air, consist of small particles named droplet nuclei (1–5μm) or droplets (>5μm). Droplet nuclei can stay airborne for hours, transport over long distances and contaminate surfaces by falling down [1]. It has been proven that droplets can contaminate surfaces in a range of 1 meter (3ft) [2]. The droplets are capable of penetrating deep into the alveoli, offering a potential route of infection [3]. The susceptibility of acquiring an infectious agent is determined by factors such as: virulence; dose; and pathogenicity of the micro-organism; and the host’s immune response [3–5]. Humans generate bio-aerosols by talking, breathing, sneezing or coughing [1]. Based on the infectious status of a person, the bio-aerosols are proven to contain influenza or rhinoviruses [6, 7], *Mycobacterium tuberculosis* [3], *Staphylococcus aureus*, Varicella Zoster Virus, *Streptococcus* spp. or *Aspergillus* spp. [8]. Moreover, bio-aerosols can be generated by devices such as ventilation systems, showers and high energetic instruments running on tap water. Showers and instruments cooled with tap water are able to spread environmental microbes such as *Legionella* spp. or other bacteria originating from water sources or water derived biofilms from tubing [4, 5, 9].

Due to the nature of their profession, healthcare workers (HCWs) are at higher risk to acquire pathogenic micro-organisms. Their risk of exposure is in line with the infectious nature of their patients, interventions or instruments that produce bio-aerosols. HCWs working in wards with patients suffering from pneumonia, who produce high virulence bio-aerosols, or HCWs exposed to bio-aerosol sources in dental practices, are at higher risk for developing disease or allergies [10, 11]. According to a risk assessment study, conducted in a hospital with HCWs exposed to high risk procedures, a risk ratio (RR) of 2.5 was found for acquiring viral or bacterial infection [12]. Multiple studies have found that HCWs were at higher risk to acquire an infectious disease, observing a high serological status of *Legionella* spp. and high rates of asymptomatic tuberculosis in dental practitioners and hospital staff [10, 13–15]. It is plausible that other diseases could also be acquired via bio-aerosols. Finally, evidence shows that patients with cystic fibrosis, who are immunosuppressed, are highly susceptible to airborne agents like *Pseudomonas* spp. [16]. Knowing this, we can assume that bio-aerosols with a high load of micro-organisms are a threat to immunocompromised patients suffering from leukemia, psoriasis, aplastic anemia and others [17]. Thus, the risk of acquiring pathogenic agents by bio-aerosols may be a hazard to both healthy and immunosuppressed patients as well as to HCWs.

To our knowledge, no detailed summary of the evidence regarding bio-aerosols in dental and hospital settings is available. Therefore, we chose to perform a scoping review on the present body of evidence regarding bio-aerosols. This results in an up-to-date summary of the literature, allowing us to make recommendations for future research by identifying gaps in current knowledge, and to underline the risks for HCW and immunocompromised. Since this is a scoping review, our objectives are broad and cover three areas concerning bio-aerosols in hospital and dental settings [18, 19]:

- What are the sources that generate bio-aerosols?
- What is the microbial load and composition of bio-aerosols and how were they measured?
- What is the hazard posed by pathogenic micro-organisms transported via the aerosol route of transmission?

## Methods

### Design and search strategy

A scoping review was performed systematically according to the PRISMA statement for transparent reporting of systematic reviews and meta-analysis [20] and JBI Briggs Reviewers Manual [21] (see S1 PRISMA checklist). Three search strings were run in PubMed and EMBASE from inception to 09-03-2016. In PubMed the following strings were combined: Hospitals [Mesh] OR hospital OR hospitals OR "health care category" [Mesh] OR "health care" OR "Cross infection" [Mesh] OR "cross infection" OR cross-infection OR nosocomial OR "health facilities"[Mesh] OR "health facility" OR "health facilities" AND aerosols [Mesh] OR aerosol OR aerosols OR bioaerosol OR bio-aerosol OR "bio aerosol" OR bio-aerosols OR "bio aerosols" AND bacteria [Mesh] OR bacteria OR bacterial OR bacteremia OR bacteraemia OR sepsis OR septicaemia OR septicemia OR virus OR viruses OR viral OR viridae OR viral OR viruses [Mesh] OR Amoebozoa [Mesh] OR amoebozoa OR amoebe OR amoebas OR amoebic OR fungi [Mesh] OR fungus OR fungal OR fungi OR fungating OR parasites [Mesh] OR parasitic OR parasite OR parasites OR parasitemia OR parasitemias OR “micro organism” OR “micro organisms” microorganism OR microorganisms OR micro-organism OR micro-organisms OR “health care associated infections” OR infections OR infection OR infectious. For EMBASE we used the following strings combined: ‘hospitals/exp OR hospitals OR (health AND care AND category) OR healthcare OR ‘cross infection’OR ‘health facility’ OR ‘health facilities’ AND Infection/exp OR microorganism/exp OR fungi/exp OR virus/exp OR sepsis/exp OR bacteria/exp AND Aerosol OR aerosols/exp OR bioaerosol OR bio-aerosol OR bioaerosols OR aerosols.

### Screening process and inclusion criteria

References yielded from the search strategy were imported in Covidence, an online web application for screening systematic reviews, and duplicates were removed. C.Z. and A.L. screened and scored the relevance of the hits independently, based on their title and abstract. The full text manuscripts were retrieved via Endnote, Google, Research Gate or by addressing the corresponding and/or first author. Subsequently, the studies were assessed on their eligibility for inclusion based on the full text. A study was included for final data extraction and summary when it met one of the following criteria: bio-aerosol composition; pathogenicity; sources; conducted in healthcare or the dental setting; published in English, German, French, Spanish or Dutch. Discussion papers, letters to the editor, animal studies, protocols, prevention of bio-aerosols, technical studies, reviews without pooled data, narrative reviews, development of drug therapy, or studies conducted in other settings besides healthcare were excluded. Additionally, a reference check and search through grey literature was conducted and included in the flowchart termed ‘snowballing’.

### Data extraction and summary

Data on the origin of bio-aerosols was categorized based on sources. Studies on the microbial composition of the bio-aerosols were summarized based on the colony forming units (CFU). References that reported sampling time were recalculated for a sampling time of 10 minutes and finally Log-transformed to make comparison possible between studies. These studies are presented in figures. References not reporting sampling time were not summarized and are presented in the study of characteristics table. The micro-organisms reported in individual studies were summarized per type of organism and setting. Potential hazard for patients and HCWs were summarized narratively.

## Results

**Fig 1:**
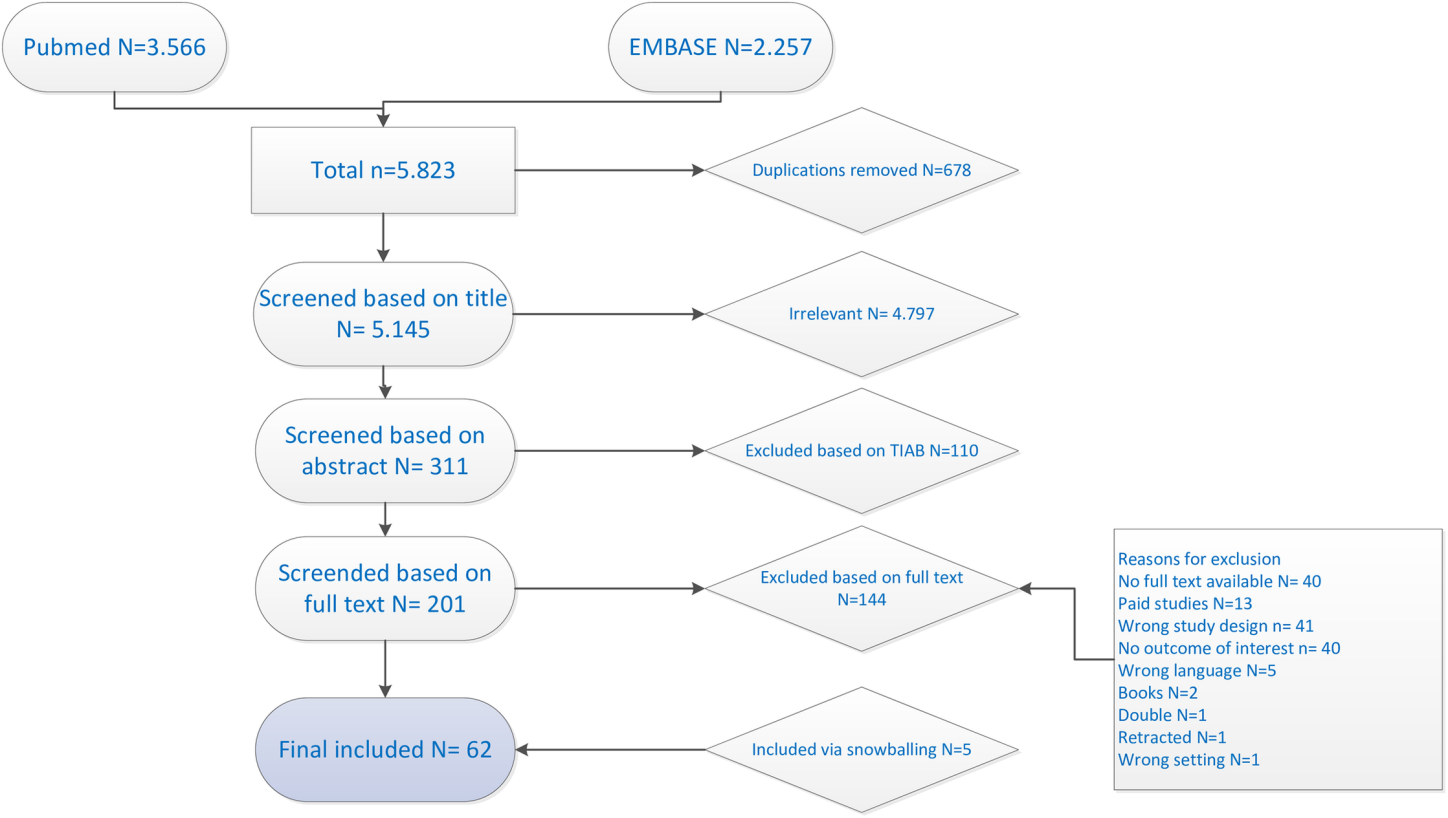
PRISMA flowchart.

A total of 5823 studies were retrieved, of which 678 duplicates and 4797 irrelevant studies were removed. After reading 311 abstracts, 201 full text studies were assessed for eligibility. This eventually resulted in 62 studies including references from snowballing (see Fig 1. PRISMA flowchart).

### Generation of bio-aerosols

One study reported solely on the generation of bio-aerosols [22]. Therefore, we extracted data on the generation of bio-aerosols from papers selected for the other objectives [23–44]. The sources of bio-aerosols in dental clinics were: ultrasonic scalers, high speed hand pieces, air turbines, three in one syringes, and air water syringes. Studies conducted in hospitals reported 30 different bio-aerosol generating sources. Humans produced aerosols by coughing, and sneezing. Patients with cystic fibrosis positive for *Burkoderia cepacia* were also capable of producing pathogenic aerosols. Interventions conducted by HCWs that produced aerosols were: colonoscopy, tracheal intubation, suction before and after intubation, manipulation oxygen mask, bronchoscopy, non-invasive ventilation, insertion of nasogastric tube, defibrillation, chest physiotherapy, and washing the patient. Bed making, ward rounds, tea trolley round, activity at bed, floor mopping, moving furniture, lunch time, drugs round, evening meal, vacuum cleaner, toilet use, cold-mist humidifier, shower, cleaning patients room and the nebulizer were found to be other activities in a hospital to produce aerosols [22].

### Hospital environment

Thirty-one studies analyzed the microbial composition of bio-aerosols in the hospital environment [11, 30, 35–37, 39, 41–65]. The studies combined identified a total of 111 organisms by using culture techniques (see Table 1 for overview of micro-organisms identified and Table 2 study characteristics hospital setting). Fifty-six bacterial species (23 Gram-negative and 32 Gram-positive; 1 mycobacteria), 45 fungal genera and ten viral species were identified[11, 30, 35–37, 39, 41–52, 54, 56–66] Most bacteria originated from human skin or the human gut, the environment or water. The identified viruses originated from the human respiratory tract. The methods for collecting air samples from the bio-aerosols and the methods for culturing micro-organisms were heterogeneous. The method most frequently used to actively collect micro-organisms was the Andersen air sampler (N = 9). Four studies used passive collection of micro-organisms by placing Petri dishes with agar. In all studies, 21 different culture methods were used, wherefrom Tryptic soy agar (N = 7) was most frequently used.

**Table 1:** Overview of micro-organisms identified in hospital setting.

Bacteria N = 56				
Gram negative		Gram positive		
*Achromobacter xylosoxidans*	*Moraxella*	*Bacillus cereus*	*Rodococcus* spp	*Staphylococcus saprophyticus*
*Acinetobacter baumannii*	*Neisseria* spp	*Bacillus* spp	*Staphylococcus aureus*	*Staphylococcus schleiferi*
*Acinetobacter calcoaceticus*	*Ochrobactrum anthropic*	*Bacillus sutilis*	*Staphylococcus auriculans*	*Staphylococcus sciuri*
*Branhamella catarrhalis*	*Pantoea agglomerans*	*Brevibacterium casei*	*Staphylococcus capitis*	*Staphylococcus werneri*
*Burkholderia cepacia*	*Proteus*	*Clostridium difficile*	*Staphylococcus caprae*	*Staphylococcus xylosus*
*Enterobacter* spp	*Pseudomonas aeruginosa*	*Corynebacterium*	*Staphylococcus chromogenes*	*Sterptococcus pyogens*
*Escherichia coli*	*Pseudomonas fluorescens*	*Diphtheroid* spp	*Staphylococcus cohnii*	*Streptococcus* spp.
*Flavobacterium* spp	*Pseudomonas putida*	*Kocuria kristinae*	*Staphylococcus epidermidis*	*Viridans Streptococci*
*Klebsiella oxytoca*	*Rahnella aquatilis*	*Kocuria varians*	*Staphylococcus haemolyticus*	Other strain:*Mycobacterium tuberculosis*
*Klebsiella pneumonia*	*Shigella*	*Micrococcus luteus*	*Staphylococcus hominis*	
*Legionella pneumophila*	*Strenotrophomonas maltohilia*	*Micrococcus lylae*	*Staphylococcus lentus*	
*Neisseria flavescens*		*Micrococcus* spp	*Staphylococcus saprophyticus*	
**Viruses N = 10**				
Human metapneumovirus	Human adenovirus	Influenza A H1N1	Influenza B virus	Influenza virus
Parainfluenza virus 1–3	Picornavirus	Respiratory syncytial virus	Rhinovirus	Toque teno virus
**Parasites N = 0**				
None reported				
**Fungi N = 45**				
*Absidia* spp	*Candida* spp	*Epicoccum* spp	*Nigrospora* spp	*Scopulariopsos* spp
*Acremonium* spp	*Chaetomium* spp	*Exserohilum* spp	*Paecilomyces* spp	*Sepedonium* spp
*Alternaria* spp	*Chrysonilia* spp	*Fusarium* spp	*Penicillium* spp	*Sporotrichum*
*Aspergillosis fumigatus*	*Chrysoporium* spp	*Geotrichum candidum*	*Phoma* spp	*Stemphylium* spp
*Aspergillus flavus*	*Cladosporium* spp	*Geotrichum* spp	*Pithomyces* spp	*Syncephalastrum* spp
*Aspergillus niger*	*Conidiobulus* spp	*Gliocladium* spp	*Rhinocladiella* spp	*Trichoderma album*
*Aspergillus* spp	*Curvalaria* spp	*Monilia* spp	*Rhizopus nigricans*	*Trichosporon*
*Aureobasidium* spp	*Dactylaria* spp	*Mucor* spp	*Scedosporium* spp	*Ulocladium* spp
*Bipolaris* spp	*Emonsia* spp	*Myclia sterilia*	*Scopulariopsis brevicaulis*	*Verticillium* spp

**Table 2:** Study characteristics hospital setting.

Study	Set up	Findings
**Anderson 1996**	Setting: Pediatric hematology and oncology ward	Fungi:*Aspergillosis fumigatus*
	Sampling method: Air dust sampler L100; 100L/min; 10 min before and after vacuum cleaning; 0.5m above cleaner. Fungal isolates by morphology. Sampling: Before and after vacuuming	Before: 24 CFU/m^3^ After: 62 CFU/m^3^
**Augustowska 2006**	Setting: Pneumological ward, hospital	Mean monthly CFU/m^3^ range of airborne bacteria:257.1–436.3Mean monthly CFU/m^3^ range of fungi:9.9–96.1
	Sampling method Air sampled with a custom-designed particle-sizing slit sampler; 20L/min; daily at 9:00 and 13:00h. Blood and Sabouraud agar.	Bacteria:
		Gram positive cocci: 34.4–46.4% à *Staphylococcus epidermidis; Micrococcus; Streptococcus*.
		Gram negative: 11.8–27.5%à *Flavobacterium* spp*; Acinetobacter calcoaceticus; Pantoea agglomerans; Escherichia coli; Enterobacter* spp*; Klebsiella oxytoca; Pseudomonas auruginosa; Branhamella catarrhalis; Neisseria flavescens; Corynebacterium; Rodococcus* spp*; Bacillus* spp.Fungi: 7.6–42.5%*Aspergillus fumigatus*: 77%*Aspergillus niger; Aspergillus flavus; Aspergillus* spp*; Penicillium* spp; *Geotrichum candidum; Trichoderma album; Mucor* spp*; Rhizopus nigricans*.
**Best, 2012**	Setting: Toilet, hospital	Mean CFU:
	Sampling method: Air sampled using AirTrace Environmental portable sampler placed at toilet seat height; 250-500L, 10cm above seat and 25 cm at handle height; 28.3L/min. Selective agar plate placed around the toilet; placed before flushing and remained for 90 min.	After flushing: 36Recovery of C. difficile mean CFU:
		Toilet lid closed vs open 0–30 min: 4 vs 1630–60 min: 4 vs 360–90 min: 0 vs 1
		Bacteria:*C*. *difficile*
**Cabo Verde, 2015**	Setting: OT, SW, ES hospital	CFU/m^3^ range bacteria:ES: 240-736SW: 99-495OT: 12–170
	Sampling method: Air sampler MAS-100 100L/min; 1m above floor when possible.Trypic soy agar; malt extract agar; gram staining.	CFU/m^3^ range fungiES: 27–933 SW: 1–32 OT: 0–1
		Total % bacteria*Staphylococcus aureus*, *capitis*, *hominis*, *epidermis*, *werneri*: 51%*Micrococcus luteus*, *lylae*: 37%*Neisseria*: 4%*Brevibacterium casei*: 1%*Shigella*: 0.3%*Proteus*: 2%
		Total % fungi
		Penicillium spp: 41%*Aspergillus* spp: 24%*Cladosporium* spp: 14%*Chrysonilia* spp: 5%*Chrysoporium* spp: 3%*Scopulariopsis brevicaulis*: 3%
**Chen, 2008**	Setting: TB and non TB area; hospital	Average concentration:
	Sampling method: Air sampled using Nucleipore filter; 20L/min; 2x4h a day; 1.2–1.5m height. DNA isolation qPCR	TB area:3.8 x 10^3^ ± 1.7 x 10^3^ copy/m^3^
		Non-TB area:3.9 x 10 ± 2.1 x 10 copy/ m^3^
		Emergency department:4.0 x 10^3^ ± 2.6 x 10^3^ copy/m^3^Ward area medical department:10^2^ ± 5.5 x 10^1^ copy/m^3^
**Chen, 2007**	Setting: TB and Non TB area; Hospital	Range concentration:1.43 x 10 copy/m^3^ to 2.06 x 10^5^ copy/m^3^
	Sampling method: Air sampled using Nucleipore filter; 22L/min; 8h sampling; 1m from patients bed at 1.2–1.5m height. DNA isolation qPCR	
**Fekadu, 2015**	Setting: MW, SW hospital	Mean CFU/m^3^ (range):
	Sampling method: Air samples were taken by passive method. Petri dishes placed 1m above the floor in room’s center. Samples collected in the morning and evening time of exposure: 30, 60, 90 min. Nutrient and Sabouraud agar plates.	Bacteria: 5583 (3106–9733)
		Fungi: 3008 (2123–4168)
**Fennelly, 2012**	Setting: TB and Non TB area; Hospital	TB median CFU (range):16 (1–710)
	Sampling method: Cough bio-aerosol sampling system method used to collect bio-aerosols in TB positive patients. Andersen six-stage sampler with 7H11 agar collection for 5 minutes.	
**Humphreys, 1994**	Setting: Hospital	Bacteria:*B*. *cepacia*
	Sampling method: CF patients colonized with *B*. *cepacia*. Air sampled with Surface Air System air sampler, 900 l for 5 min; 100 cm from patient. Samples taken 15 min intervals for one hour after patient had left the room and 18 h after vacating. Selective *B*. *cepacia* agar medium.	During stay CFU/m^3^:Range: 1-158Mean: 32
		After stay CFU/m^3^:Range: 3-53Mean: NR
**Huynh, 2008**	Setting: Laboratory	Virus:Influenza virus; para influenza virus; Rhinovirus; Influenza A virus.
	Sampling method: Reading aloud (20 min), quiet breathing (20 min), 20 voluntary coughs over a 3–5 min period in a separate sampling mask. PCR	
**Heimbuch, 2016**	Setting: Hospital	Mean CFU/cm^2^Mask 1 external layer: 24.15Mask 2 external layer: 3.33
	Sampling method: No direct bio-aerosol sampling. Viable bacteria found on mask was measured. Tryptic soy agar; Gram staining.	Bacteria:*Kocuria kristinae*, *varians; Micrococcus* spp.; *Staphylococcus aureus*, *S*. *auriculans*, *S*. *capitis*, *S*. *caprae*, *S*. *chromogenes*, *S*. *cohnii*, *S*. *epidermidis*, *S*. *haemolyticus*, *S*. *hominis*, *S*. *lentus*, *S*. *saprophyticus*, *S*. *schleiferi*, *S*. *sciuri*, *S*. *warneri*, *S*. *xylosus; Acinetobacter baumannii; Ochrobactrum anthropic; Pesudomonas fluorescens /putida; Rahnella aquatilis; Stenotrophomonas matophilia*.
**Knibbs, 2014**	Setting: Children’s hospital	Total mean CFU/m^3^ (range) per distance:1m: 52.6 (40.9–67.6)2m: 37.3 (28.9–48.0)4m: 24.8 (19.2–32.0)
	Sampling method: CF patients colonized with *B*. *cepacia*. Cough bio-aerosols collected by Andersen Impactor (28.3L/min); chocolate bacitracin agar for *B*. *cepacia* and isolates by RT-PCR.	Total mean CFU/m^3^ (range) per duration:5 min: 14.6 (11.0–19.2)15 min: 11.9 (8.9–15.7)45 min: 7.7 (5.4–11.0)
		% of subjects positive for bacteria:*P*. *aeruginosa*: 100Mucoid *P*. *aeruginosa*: 78.9Non-mucoid *P*. *aeruginosa*: 94.7*S*. *aureus*: 26.3*Strenotrophomonas maltohilia*: 10.5
		Subjects positive for fungi (%):*Trichosporon*: 15.8*Aspergillus* spp: 15.8*Scedosporium* spp: 10.5*Candida* spp.: 5.7
**Kulkarni, 2016**	Setting: Infant nursing bay, hospital	Mean PFU of RSV:315.189; range: 82.600–1.120.000
	Sampling method: Air sampled with Westech six-stage Microbial sampler; 28.3L/min for 30 min; 1m distance from infected infant. PCR	2h after discharge:6.175.
		Virus:RSV
**Kumar, 2010**	Setting: GW, PW, OT, ICU, hospital	Bacteria %:*S*. *aureus*: 43.8*Escherichia coli*: 17.9*Klebsiella pneumonia*: 16*Sterptococcus pyogens*: 13.2*Pseudomonas aeruginosa*: 8.9
	Sampling method: Air sampled by passive settling plate technique. Nutrient, blood, and MacConkey’s agar 5 min exposure.	
**Lindsley, 2015**	Setting: Laboratory	Range 5 to 538 PFU, mean (SD): 142 (125).
	Sampling method: Bio-aerosols collected using SKC BioSampler with 5ml vessel containing viral transport media: Hank Balanced Salt Solution. qPCR	Virus: Influenza A
**Li, 2003**	Setting: ICU, hospital	Range of mean CFU/m^3^ Fungi: 0–3115
	Sampling method: Air was sampled using Andersen six stage sampler; 28.3L/min; 1m height. Sampling time 20–30 min. Tryptic soya and malt extract agar.	Bacteria: 0–423
**Martins-Diniz, 2005**	Setting: SW, ICU, hospital	Mean total fungi CFU/m^3^
	Sampling method: Air samples taken by Andersen sampler. 80L of air sampled on Sabouraud agar and chloramphenicol culture medium. Samples taking during the morning and immediately after cleaning, late afternoon and end of regular shift, monthly collections.	SC morning: 13,362
		SC afternoon: 20,939
		ICU morning: 16.925
		ICU afternoon: 16,392

		Total CFU/m^3^ SW & ICU morning/afternoon:
		*Cladosporium* spp.: 6,338/16,587 11,587/11,192
		*Fusarium* spp: 2,350/900 514/612
		*Pencillium* spp: 912/813 1,425/950
		*Chrysosporium* spp.: 401/562 637/950
		*Aspergillus* spp.: 362/289 775/413
		*Aureobasidium* spp.: 562/200 238/476
		*Myclia sterilia;* 350/300 64/237
		*Monilia* spp.: 325/100 62/250
		*Paecilomyces* spp.: 89/275 162/175
		*Curvalaria* spp.: 262/200 26/75
		*Chaetomium* spp.: 275/12 75/212
		*Stemphylium* spp.: 162/100 38/63
		*Rhinocladiella* spp.: 75/38 137/0
		*Exserohilum* spp.: 25/0 87/38
		*Epicoccum* spp: 0/75 88/150
		*Phoma* spp.: 100/25 201/125
		*Alternaria* spp.: 26/26 137/88
		*Nigrospora* spp.: 162/25 13/100
		*Syncephalastrum* spp.: 51/87 137/25
		*Bipolaris* spp.: 25/25 0
		*Dactylaria* spp.: 12/0 37/37
		*Acremonium* spp: 25/12 87/26
		*Conidiobulus* spp.: 0 12/112
		*Verticillium* spp.: 12/0 87/0
		*Gliocladium* spp.: 62/37 87/0
		*Pithomyces spp*.: 0/25 100/0
		*Sepedonium spp*.: 0 0
		*Scopulariopsos* spp.: 0/25 25/12
		*Sporotrichum*: 0/12 50/0
		*Ulocladium* spp.: 0/12 0/25
		*Scedosporium* spp.: 25/0 0
		*Emonsia* spp.: 0 0/12
		*Geotrichum* spp.: 12/0 0
**Menzies, 2003**	Setting: Screening center for TB	Average airborne viable CFU/m^3^ at induction:
	Sampling method: Sputum induction by ultrasonic nebulizer for 15 min. Samples taken with Andersen six stage sampler 15L/min, at the height of the therapists breathing zone (1.5m height). Sheep blood agar.	Before: 233
		During: 351
		After: 106
**Mirhoseini, 2015**	Setting: OT, ICU, SW, IM, hospital	Total mean CFU/m^3^:
	Sampling method: Air sampled with AGI 12.5L/min 180–240 min. Tryptic soy agar culture.	OT: 396
		ICU: 222
		SW: 537
		IM: 524
		Total: 420
**Mirzai, 2014**	Setting: OR, ED, Hospital	Total mean CFU/m^3^ ED & OR:
	Sampling method: Air samples taken by eight step Andersen 28.1L/min. Once a month for a year every morning before start of the shift. Plates at 1m height and 1m from obstacles exposed for 10 min. Blood, BHI, and McConkey agar.	Total ER: 103.88 (33.84)
		Total OR: 63.32 (32.94)
		Micrococcus: 14.85/16.09
		Streptococcus A: 1.26/2.19
		Viridans Streptococci: 2.92/1.72
		Pneumococcus: 7.81/3.81
		Escherichia coli: 6.91/2.0
		Bacillus sutilis: 6.64/1.63
		S. aereus: 14.17/10.92
		S.epidermis: 10.95/5.72
		S. saprophyticus: 11.35/5.45
		Bacilluscereus: 7.14/1.73
		Diphtheroid spp: 5.28/2.27
		Pseudomonas spp: 4/3.47
		Klebsiella: 4.19/1.09
		Enterobacter: 1.17/0.27
		Citrobacter: 0.77/1.62
		Branhamla: 0.19/0.36
		Bacterial, 20^°^C CFU/m^3^ mean (range):
		Conv. Flow morning: 141 (42–325)
		Conv. Flow evening: 82 (49–141)
		Lam. Flow morning: 25 (0–92)
		Lam. Flow evening: 82 (7–233)Bacterial, 30^°^C CFU/m^3^ mean (range):
		Conv. Flow morning: 49 (14–92)
		Conv. Flow evening:77 (35–170)Lam. Flow morning: 110 (14–572)
		Lam. Flow evening: 11 (0–42)Gram negative CFU/m^3^ mean (range):
		Conv. Flow morning:2 (0–14)
		Conv. Flow evening: 0
		Lam. Flow morning: 0
		Lam. Flow evening: 0Fungi CFU/m^3^ mean (range):
		Conv. Flow morning: 22 (0–71)
		Conv. Flow evening: 38 (0–99)
		Lam. Flow morning: 5 (0–21)
		Lam. Flow evening: 80 (0–455)
**Ortiz, 2009**	Setting: General hospital	Average 2 years TAC:
	Sampling method: Air sampler MAS-100 100L/min; petri dish. Total aerobic count; rose-bengal agar, microscopy and staining.	OT: 25.6 CFU/m^3^
	Sampling: Before and after intervention. 2 years. Plate count agar; Rose Bengal agar	MW: 67 CFU/m^3^
		HR: 124.4 CFU/m^3^ Average 2 years FL:
		OT: 0.05 CFU/m^3^
		MW: 6.9 CFU/m^3^
		HR: 10.6 CFU/m^3^ Fungi:
		*Aspergillosis fumigatus*, *flavus*: 89%, 11%.
		Bacteria:
		not specified.
**Sudharsanam, 2012**	Setting: OW, Hospital	Total range of bacteria:
		45–150 CFU/plate.
	Sampling method: Passive air sampling and active by active using filter and impinger. Petri dishes at 60–70 cm height, exposed for 30 min. 3.5L/min of air for 20 min. Samples taken in multiple months. Blood, Sabouraud’s Dextroser, and MacConkey agar.	Total range of fungi: 0–13 CFU/plate
		Bacteria:
		Coagulase negative staphylococci; micrococci; *Enterobacter; Pseudomonas*.
		Fungi:
		*Aspergillus fumigatus; flavus; niger*.*; Absidia* spp.
**Stelzer-Braid, 2009**	Setting: Hospital	Virus:
	Sampling method: Bio-aerosols collected with face mask. RT-PCR	Influenza A; Influenza B; parainfluenza 1, 2, 3, respiratory syncytial virus, human metapneumovirus, picornavirus.
**Thompson, 2013**	Setting: Hospital	Median RNA copy no/L (IQR): Airway suction: 1.852 (1.543–2.7521)
	Sampling method: Air sampled with Glass May-3stage impingers at 55L/min at 1m height; 1m from patients head; 40 min collection. RT-PCR.	Baseline: 7.913 (2.436–11.613)
		Bronchoscopy: 148.805 (12.735–284.875)
		Intubation: 2.838 (2.838–2.838)Virus:
		Influenza A H1N1
**Vavricka, 2010**	Setting: Endoscopy unit, hospital	Mean CFU/m^3^ (range) with air suction:
	Sampling method: Air sampled with MAS-100; 100L/min; before first colonoscopy and end of evening; 30 sec; 1.2m height; 0.3m distance. Colombia agar.	Before: 4 (1–7)
		End: 16 (9–22)
		Mean CFU/m^3^ (range) without air suction:
		Before: 14 (0–142)
		End: 7 (1–88)Bacteria:
		*Staphylococci* spp.
**Vélez-Pereira, 2014**	Setting: ICU, hospital	Range mean (SD) CFU/m^3^:
	Sampling method: Air sampled with cascade impactor; 28.3L/min; 1.5m height; mannitol salt; pseudomonas agar.	*Staphylococcus* spp 67.3 (1.7)– 277 (59.2)
		*Pseudomonas* spp 6.4 (1.7)– 204 (9.2)
		Bacteria %:
		*Staphylococcus* spp.: 71.5
		*Pseudomonas aeruginosa*: 64.6
**Verani, 2014**	Setting: Nephrology, hospital	Mean concentration/cm^2^ (SD):
	Sampling method: Air sampled with microflow impactor sampler; 1,000L for virus and 180L for bacteria sampled; tryptone soy agar; samples before and after disinfection toilet. RT-PCR	Human adenovirus
		Before disinfection: 349 (51)
		After disinfection: 1,371 (49)
		Total viruses %:
		Human adenovirus: 77
		Toque teno virus: 15
		Norovirus: 0
		Combination: 7
		Total bacteria %: 41
		Before disinfection: 1.57
		After disinfection: 1,371 (49)
**Wainwright, 2009**	Setting: Children’s Hospital	Voluntary cough range CFU: 0–13,485
	Sampling method: Air sampled with Cough bio-aerosol sampling system and Anderson six stage impactors for 5 min in children with CF positive for *Pseudomonas aeruginosa*. Chocolate agar.	Mean CFU settle plate: 6 (95%CI 3–14)
		Bacteria:
		*Pseudomonas aeruginosa; Strenotrophomonas maltophilia; Achromobacter xylosoxidans*.
**Wan, 2011**	Setting: OR, hospital	Median CFU/m^3^ (range):
	Sampling method: Air sampled with Andersen one-stage viable impactor at 28.3L/min volume for 3 min; 1.2–1.5m height; 1.5m from operation tables. Tryptic soy agar.	Transplantation room: 76.0 (9–477)
		Trauma room: 121.5 (13–756)
		Cardiovascular surgery: 13 (0–163)
		Colon surgery: 73.5 (0–567)
		Orthopedic surgery: 89 (4–243)
		Bacteria % in transplantation, trauma, cardiovascular surgery, rectal and orthopedic surgery room:
		*Bacillus* spp: 32; 24; 16; 16; 34
		*Micrococcus* spp: 35; 25; 26; 39; 9
		*Staphylococcus* spp: 16; 20; 33; 26; 23
		*Acinetobacter* spp: 0; 8; 3; 0; 6
		*Moraxella* spp: 1; 0; 6; 0; 0;
		*Pseudomonas* spp: 1; 0; 0; 0; 0
**Woo, 1986**	Setting: Hospital	Distance (cm) and colonies/plate:
	Sampling method: Air sampled by passive technique placing plates in different places within a hospital shower. Buffered charcoal yeast extract agar.	Adaptor / adaptor + extension:
		0: 3 / 3
		1: 10 / 5
		4: 7 / 5
		10: 4 / 2
		20: 2 / 1
		Bacteria:
		*Legionella pneumophila*

Abbreviation: AMHB = aerobic mesophilic heterotrophic bacteria; BHI = blood heart infusion; CFU = Colony forming units; CF = cystic fibrosis; cm = centimeters; ED = emergency department; ER = emergency room; ES = emergency service; FL = fungal load; GW = general ward; HR = hospital room; IM = internal medicine; IQR = inter quartile range; L = liters; min = minutes; m = meters; MW = maternity ward; NR = not reported; NTM = nontuberculous mycobacteria; OR = operation room; OT = operation theatre; OW = operation ward; PFU = plaque forming units; PW = pulmonary ward; RSV = respiratory syntical virus; SC = surgical center; SD = standard deviation; SW = Surgical Ward; TAC = total aerobic count; TB = tuberculosis; qPCR = quantitative polymerase chain reaction.

**Fig 2:**
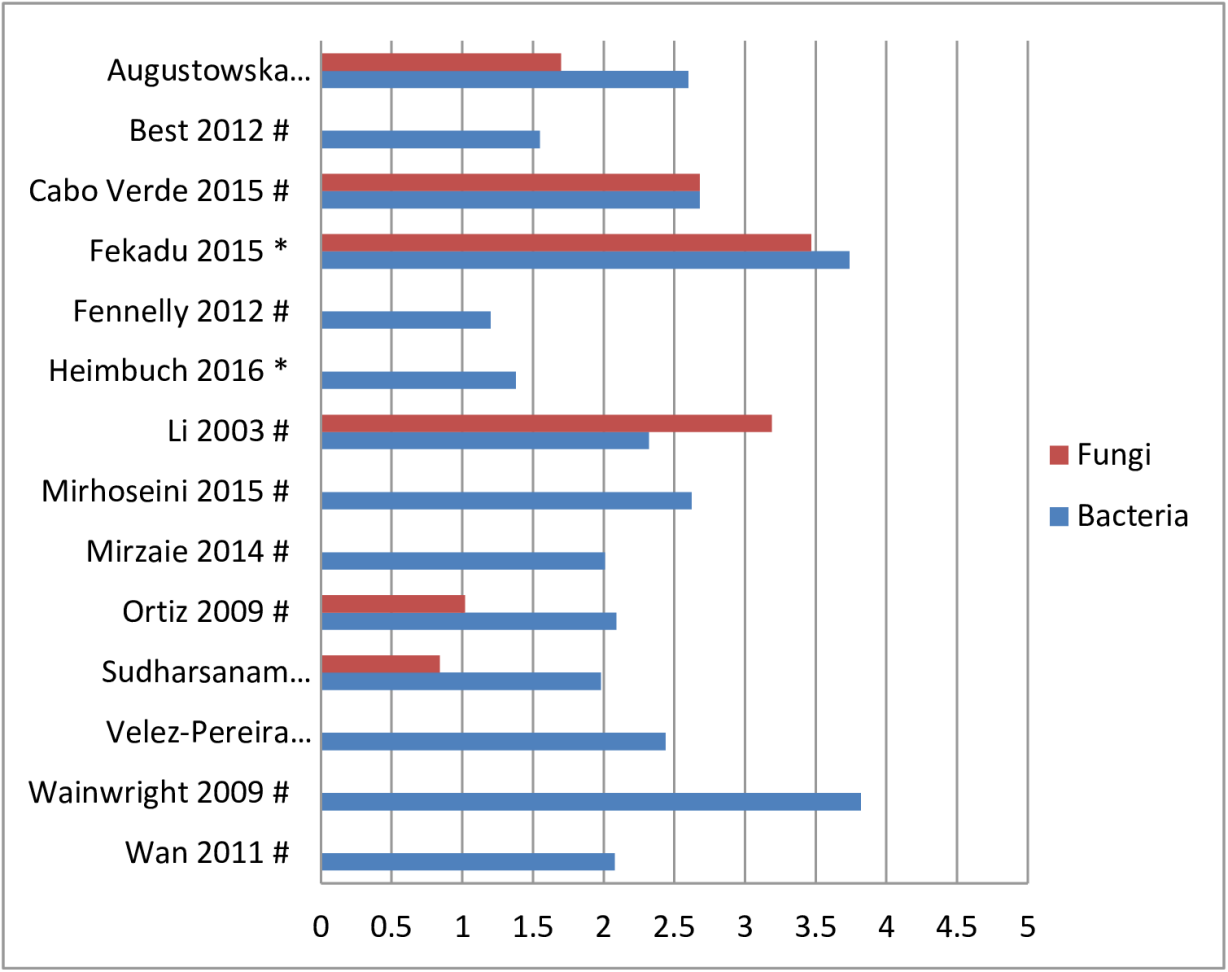
Bacterial or fungal loads in mean Log-10 CFU/m^3^ in hospitals. * = passive sampling method; # active sampling method.

**Fig 3:**
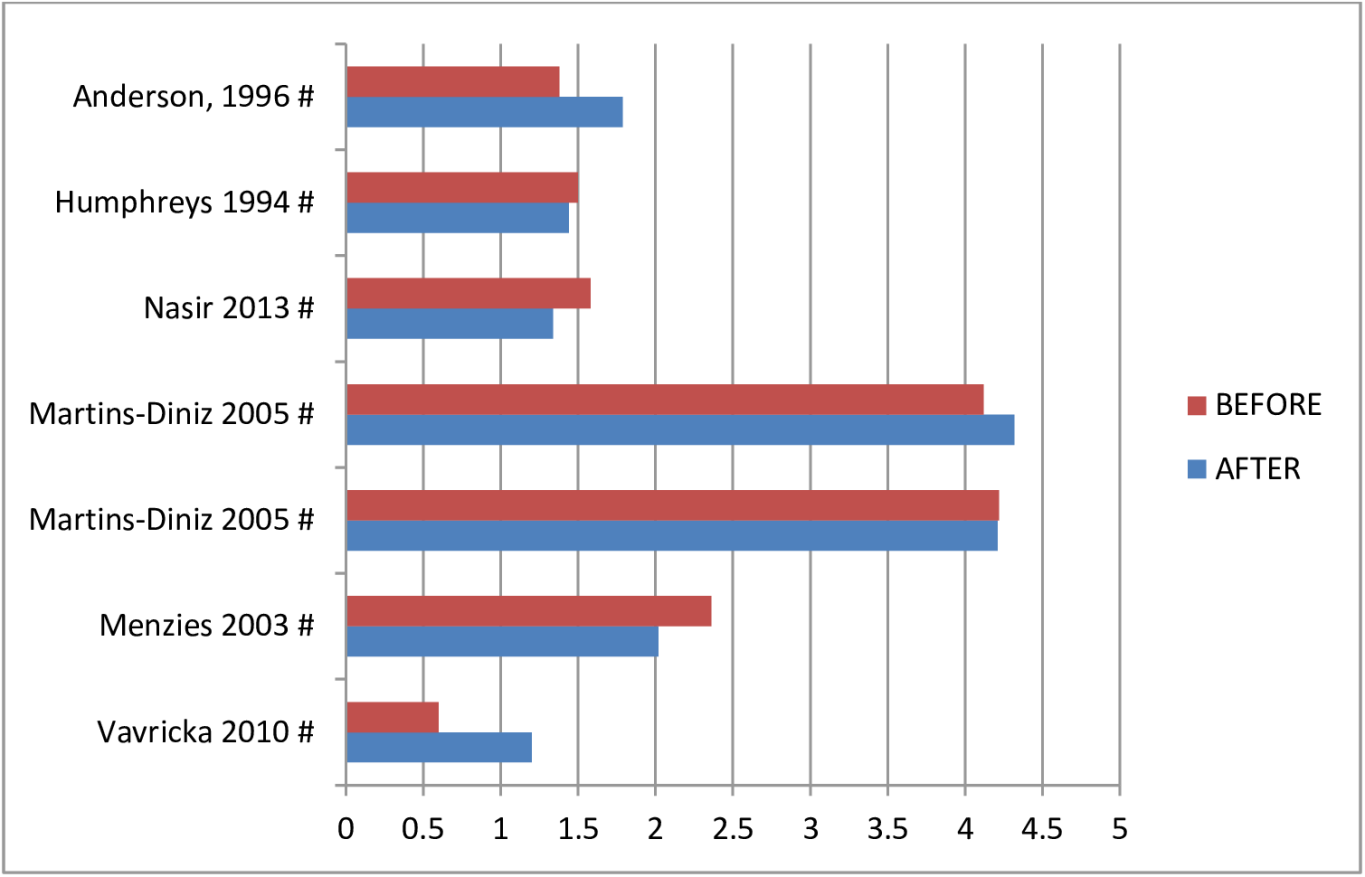
Bacterial or fungal loads in mean Log-10 CFU/m^3^ in hospitals, measured twice. * = passive sampling method; # active sampling method.

Fourteen studies analyzed the bacterial load of the bio-aerosols [11, 39, 41, 42, 45–47, 50, 55, 58, 60, 61, 64, 65]. The mean Log-10 of CFU/m^3^ ranged from 0.8 to 3.8 (see Fig 2). Additionally, five studies analyzed the bio-aerosol contamination before and/or after treatment, intervention or of a room when a patient with an infectious disease was present. The measured bacterial or fungal load ranged from Log 0.6–4.2 at baseline to Log 1.2–4.3 after the second measurement (see Fig 3) [30, 35, 43, 56, 57]. Seven studies reported on the fungal load in bio-aerosols during the day when patients were present in a hospital room. Fungal loads ranged from Log 0.8–3.5 CFU/m^3^ in various hospital wards [45, 47, 50, 56, 59, 61, 66]. Multiple studies quantified the air in patient specific areas or via specific methods.

Two studies identified multiple viruses in bio-aerosols after patients with symptoms of a cold coughed, however both studies did not report on the viral load [51, 62]. Viral loads in the bio-aerosol ranged between Log 2.2 plaque forming units /m^3^ in the air of an infant nursery positive for RSV and Log 5.5 PFU/m^3^ in the air contaminated by patients positive for Influenza A virus [52, 54]. Another study reported the RNA copy/L and found Log 3.3–5.2 in aerosols produced by patients positive for Influenza A virus [33].

### Dental environment

Seventeen studies analyzed the microbial composition of dental clinics [23–29, 67–76]. The studies cumulatively identified 38 types of micro-organisms by using culture techniques (see Table 3 for complete overview of micro-organisms identified and Table 4 for study characteristics in dental setting). Wherefrom nineteen bacteria (7 Gram-negative and 12 Gram-positive) and 23 fungal genera were detected. The bacteria originated from water, human skin and the oral cavity. None of the included studies looked for viruses or parasites. Similar to the hospital setting, the active Andersen air sampler (N = 4) and the passive culturing method by placing Petri dishes with agar (N = 6) were the most frequent used air sampling techniques. Thirteen different culture methods were used to identify the collected micro-organisms, of which Tryptic soy agar (N = 3) and blood agar (N = 3) were used most often.

**Table 3:** Complete overview micro-organisms identified in the dental setting.

Bacteria N = 19					
Gram negative		Gram positive			
*Acinetobacter wolffii*		*Staphylococcus capitis*	*Staphylococcus chromogenes*	*Micrococcus luteus*	*Diphteroids*
*Legionella* spp.		*Staphylococcus lentus*	*Staphylococcus haemolyticus*	*Micrococcus* spp.	*Corynebacteria*
*Pseudomonas aureus*		*Staphylococcus xylosus*	*Staphylococcus epidermidis*	*Micrococcus lylae*	*Bacillus* spp.
*Staphylococcus aureus*			*Staphylococcus fominis*	*Bacillus pumilus*	*Actinomycetes*
**Viruses N = 0**					
None reported					
**Parasites N = 0**					
None reported					
**Fungi N = 23**					
*Alternaria alternata*	*Aspergillus flavus*	*Cladosporium cucumerinum*	*Geotrichum* spp	*Stemphylium* spp	
*Alternaria brassicicola*	*Aspergillus fumigatus*	*Cladosporium ramotenellum*	*Monocillim indicum*	*Stemphylium* spp	
*Alternaria citri*	*Aspergillus niger*	*Cladosporium sphaerospermum*	*Monodictys glauca*	*Ulocladium alternariae*	
*Arthrinium phaesospermum*	*Botrytis* spp	*Cladosporium* spp	*Pencillium* spp		
*Aspergillus*	*Cladosporium cladosporiodias*	*Cladosporium spongiosum*	*Penicillium chrysogenum*		

**Table 4:** Study characteristics in the dental setting.

Study	Set up	Findings
**Almaghlouth, 2007**	Setting: Dental clinic	Before: *Diphteroids* spp*; Micrococcus* spp
	Sampling method: Passive air sample: Blood agar and Brain Heart agar culture plates. 15–20 min exposure during; 30 min in advance and 2h after treatment.	During: *Diphteroids* spp*; Micrococcus* spp*; Staphylococcus epidermidis*.
		After: *Diphteroids* spp*; Micrococcus* spp*; Staphylococcus epidermis*, *aureus*.
**Bennett, 2000**	Setting: Dental clinic	Range max peak CFU/m^3^
	Sampling method: Air sampled with The Casella slit sampler; 30L/min; 2 min during treatment; 1m from patients mouth at bench height. Andersen sampler 28L/min 5 min; during treatment; 2m from patient; at foot of dental chair. Tryptone yeast extract Cystine agar; Columbia blood agar.	Winter: 6.3 x 10^3^–8.7 x 10^3^
		Summer: 3.0 x 10^3^–6.2 x 10^3^
		Bacteria:
		EPS producing streptococci: 50%
**Barlean, 2010**	Setting: Dental clinic	Mean (range) CFU/m^3^ mesophilic bacteria:
	Sampling method: Passive air sample: Tryptone soy agar, sheep blood; Sabouraud agar; exposed 15 min; 30cm and 2.5m distance from dental unit.	Before: 129 (42–273)
		After: 429.6 (105–1018)
		Range CFU/m^3^ fungi:
		Before: 21–29
		After: 52–808
		Bacteria:
		Mesophilic bacteria: *Staphylococcus aureus*: 6.6.%
		Fungi: NR
**Cellini, 2000**	Setting: Dental clinic	CFU/m^3^ range:
	Sampling method: Air sampled with Air Microbial index, plate method; placed prior to exposure; 1h exposure at 1m height and 1m from wall; before and after	4–18
**Dutil, 2007**	Setting: Dental clinic	Mean endotoxin in air CFU/m^3^:
	Sampling method: Andersen six-stage air sampler; 28.3L.min during 20 min; 2h before; during and 2h after dental treatment. 30cm from patients mouth. R2A and blood agar.	Before: 0.34
		During: 0.54
		After: 0.33
**Duell, 1970**	Setting: Dental clinic	TB (not found)
	Sampling method: Andersen six stage air sampler; petri dishes; 30 cm from patient; air sampling 15min.	
**Grenier, 1995**	Setting: Dental clinic, closed and multichair	Mean CFU/m^3^ (SD) 1:
	Sampling method: Air sampled with Slit-to-agar biological air sampler, 20L/min; 30 min before, during, after treatment duration of 30 min; 122cm from patient at 91 cm height	Before: 12 (4)
	Three settings:	During: 216 (75)
	1. Closed dental operatory using ultrasonic scaler.	After: 44 (14)
	2. Operative treatments in closed dental operatory.	2h after: 10 (1)
	3. Multichair	4h after: 6 (2)
		Mean CFU/m^3^ (SD) 2:
		Before: 14 (4)
		During: 75 (22)
		After: 51 (22)
		2h after: 12 (4)
		4h after: 9 (4)
		Mean CFU/m^3^ (SD NR!)3:
		Before: 13.5
		After: 97.75
		3h after initiation: 82.75
		7h after: 9
**Guida, 2012**	Setting: Dental clinic within a hospital	Active sampling CFU/m^3^ (SD)
	Sampling method: Air sampled using Surface Air System; 180L/min 500L volume; 1m height; 1m from unit. Passive sampling with petri dish exposed for 1h. Before; during and after treatment. Tryptone soya agar.	Before: 86.6 (50.6)
		During: 82.3 (48.3)
		After: 86.5 (64.8)\
		Passive sampling CFU/m^3^ (SD):
		Before: 38.3 (21)
		During: 39.6 (28.2)
		After: 20 (11.5)
**Kadaifciler, 2013**	Setting: Dental clinic	AMHB range mean CFU/m^3^ (SD):
	Sampling method: Air sampled with HiAirflow, 100L/min, 1.4 height, near to dental unit, before; during and after treatment. Trypton soya agar for AMHB; Sabouraud and RB-agar for fungi.	Before treatment: 10 (0)– 453 (21)
		After treatment: 10 (0)– 2.477 (57)
		Fungi range mean CFU/m^3^ (SD):
		Before treatment: 0–42 (23)
		After treatment: 0–94 (95)
		Yeast range mean CFU/m^3^ (SD):
		Overall: 3–25
		Bacteria:
		*Micrococcus* spp.*; Staphylococcus xylosus; Staphylococcus lentus; Staphylococcus chromogenes*.
		Fungi %:
		NSF: 27.30 *P*. *chrysogenum*: 21.27; *Pencillium*: 11.11; *C*. *Cucumerinum*: 5.55; *Alternaria brassicicola*: 4.96; *Cladosporium* spp: 4.49; *Aspergillus flavus*: 3.86; *Alternaria alternata*: 2.83; *Alternaria citri*: 2.60; *C*. *Cladosporiodias*: 2.48; *C*. *spongiosum*: 2.48; *Aspergillus*: 1.93 *Aspergillus niger*: 1.30; *Ulocladium alternariae*: 0.82; *Arthrinium phaesospermum*: 0.82; *Stemphylium* spp.: 0.82; *Acremonium zonatum*: 0.82; *Botrytis* spp.: 0.82; *Cladosporium sphaerospermum*: 0.82; *Monocillim indicum*: 0.82; *Cladosporium ramotenellum*: 0.82; *Monodictys glauca*: 0.82.
		Yeast %:
		*Geotrichum* spp: 12.62
**Kimmerle, 2012**	Setting: Dental clinic, multi chair	Bacteria average CFU/m^3^:
	Sampling method: Air sampled with Andersen biological air sampler total of 100L at different time points. Colombia blood agar plates and enterococci-selective bile esculin agar; 16S RNA gene sequencing; Gram staining.	*Micrococcus luteus*: 188.8; *Staphylococcus epidermidis & haemolyticus*: 114.5; *Micrococcus lylae*: 16.6; *Pseudomonas*: 10.6; *Chrysemona luteda*: 0.5; *Staphylococcus hominis*: 9.0; *Acinetobacter wolffii*: 5.1; *Pseudomonas stutzeri*: 0; *Staphylococcus capitis*: 3.7; *Bacillus pumilus*: 6.8Fungi %:
		*Aspergillus fumigatus*: 4.8; *Aspergillus niger*: 0.9; *Aspergillus flavus*: 0.2
**Kedjarune, 2000**	Setting: Multi chair dental clinic	Total bacteria
	Sampling method: Air sampled with Slit-to-agar air sampler 75cm height and 30-35cm from dentists.	Total mean (SD) CFU/m^3^:
		Before: 177.41 (211.14); During: 232.49 (163.35)
		After: 44.58 (46.82)
		Total mean (SD) before/ during CFU/m^3^:
		Endodontic: 264.16 (143.53) / 270.29 (158.33)
		Operative: 188.28 (114.74) / 186.23 (69.26)
		Scaling: 245.10 (134.55) / 182.57 (63.99)
		Bacillus:
		Total mean (SD) CFU/m^3^:
		Before work: 10.89 (9.9); during work: 9.84 (20.14); after work: 3.34 (7.41)
		Total mean before and during (SD) CFU/m^3^:
		Endodontic: 11.15 (9.39) / 6.32 (4.98)
		Operative: 16.20 (32.39) / 6.17 (7.58)
		Scaling: 17.05 (11.74) / 8.69 (4.78)
**Pankhurst, 1995**	Setting: Dental clinic	Bacteria:
	Sampling method: CF patients colonized with *B*. *cepacia*. Air sampled before, during and after treatment by Surface Air System sampler, 540L per patient, 0.25m from patient. Selective culture for *B*. *cepacia*.	*B*. *cepacia* (not found)
**Pasquarella, 2012**	Setting: Dental clinic	Total mean bacteria CFU/m^3^before and after treatment:
	Sampling method: Passive air sample placing Tryptone Soya Agar for 1 hour, 1 m above the floor. Active air sampling using SAS sampler, 180L/min volume 500 L.	Passive sampling: 78/110Active sampling: 12/14
**Rautemaa, 2006**	Setting: Dental clinic	High speed:
	Sampling method: Passive air sample: Horse blood chocolate agar; 6 locations; 0.5m – 2m from patient. Gram staining. Sampling 1.5 and 3 hours exposure at start of treatment	<1m: mean CFU 823/m^2^
		>1.5m: mean CFU 1120/m^2^Periodontal: mean CFU 598/m^2^
		Bacteria:
		Gram positive cocci; Gram negative cocci; Gram positive rods; Gram negative rods.
**Szymanska, 2008**	Setting: Dental clinic	Total bacteria % before disinfection:
	Sampling method: Air samples collected with portable Air Sampler RCS plus, placed 25 cm distance from patient, 100L air per sample. Tryptic soy agar and GP2 microplate. Sampling before and after disinfection (H_2_O_2_) of DUWL.	Gram negative: 1.31; Staphylococci and other catalase positive cocci: 15.70; Streptococci and other catalase negative cocci: 79.23; Endospore forming bacilli: 1.45%; *Corynebacteria* and related organisms: 2.30; *Actinomycetes*: 0.01%
		Total bacteria % after disinfection:
		Gram negative: 1.08; Staphylococci and other catalase positive cocci: 61.19; Streptococci and other catalase negative cocci: 24.28; Endospore forming bacilli: 7.92; *Corynebacteria* and related organisms: 4.18; *Actinomycetes*: 1.35
		Mean CFU/m^3^ before disinfection:
		Gram negative: 52; Staphylococci and other catalase positive cocci: 624; Streptococci and other catalase negative cocci: 480; Endospore forming bacilli: 50; *Corynebacteria* and related organisms: 30; *Actinomycetes*: 0
		Mean CFU/m3 after disinfection:
		Gram negative: 0; Staphylococci and other catalase positive cocci: 640; Streptococci and other catalase negative cocci: 90; Endospore forming bacilli: 50; *Corynebacteria* and related organisms: 30; *Actinomycetes*: 0
**Timmerman 2003**	Setting: Dental clinic	Total CFU during high volume evacuation:
	Sampling method: Passive air sampling by placing Petri dishes during ultrasonic scaling. Baseline measure for 10min exposed dishes in middle of operatory. 2 plates on tray table over patient’s chest 40cm away from mouth. 2 plates on cart 150cm from patient’s mouth next to wall, behind patient and dentist at 100cm height exposed for 20min. BHI agar.	Before: 0.2 (0.4)
		0–5 min 40cm: 0.4 (0.7)
		20–25 min 40cm: 1.6 (1.3)
		0–20 min 150cm: 5.4 (8.3)
		20–40 min 150cm: 2.7 (3.2)
		0–40 min 150cm: 8.1 (11.3)
		Total CFU during conventional dental suction:
		Before: 0.6 (0.7)
		0–5 min 40cm: 2.5 (2.9)
		20–25 min 40cm: 1.8 (1.6)
		0–20 min 150cm: 4.3 (3.5)
		20–40 min 150cm: 6.3 (5.9)
		0–40 min 150cm: 4.0 (4.1)

Abbreviations: BHI = brain heart infusion; TB = tuberculosis; CFU = colony forming units; cm = centimeters; DUWL = dental unit waterline; h = hours; m = meters; min = minutes; SD = standard deviation.

**Fig 4:**
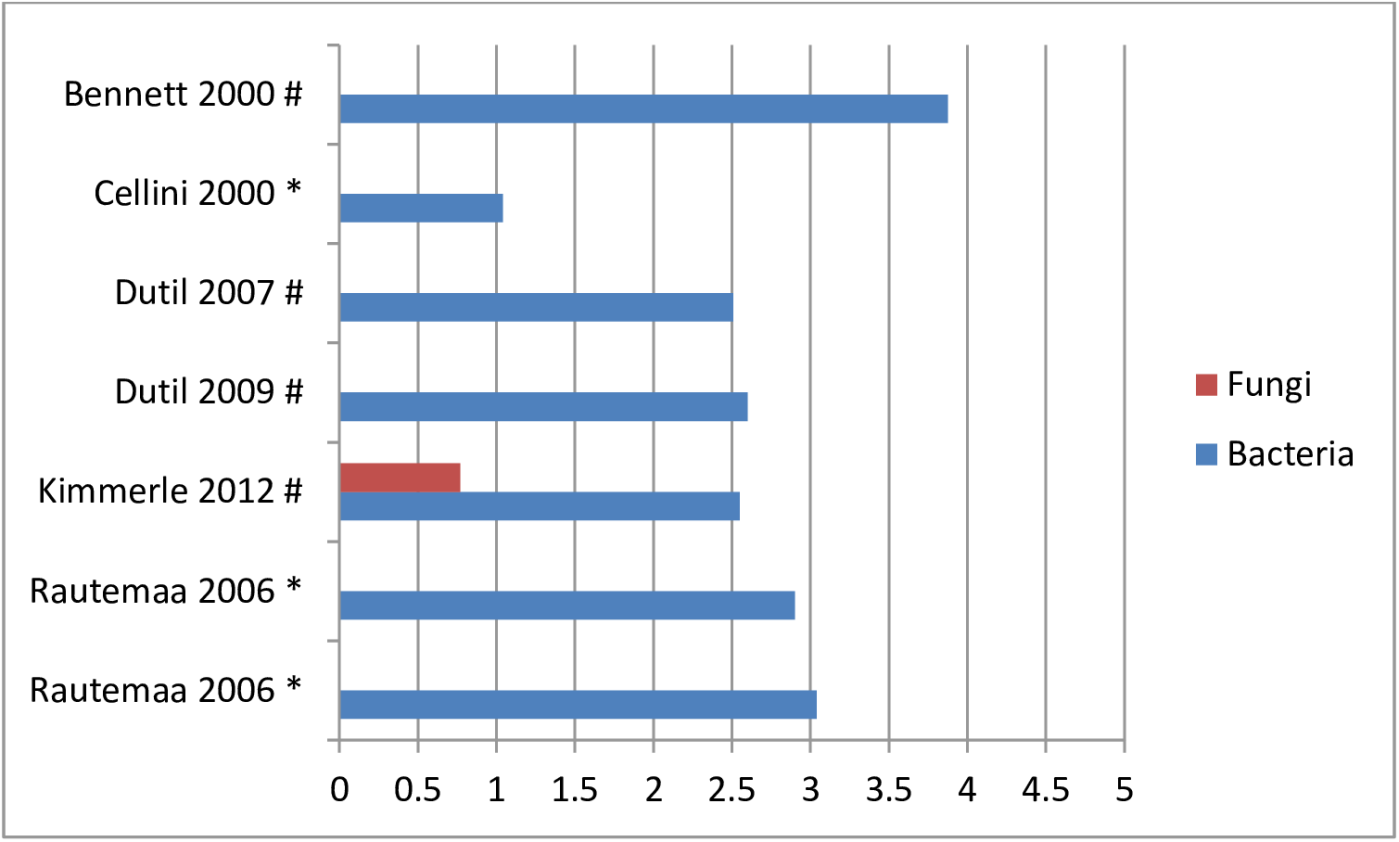
Bacterial or fungal loads in mean Log-10 CFU/m^3^ in dental. * = passive sampling method; # active sampling method.

**Fig 5:**
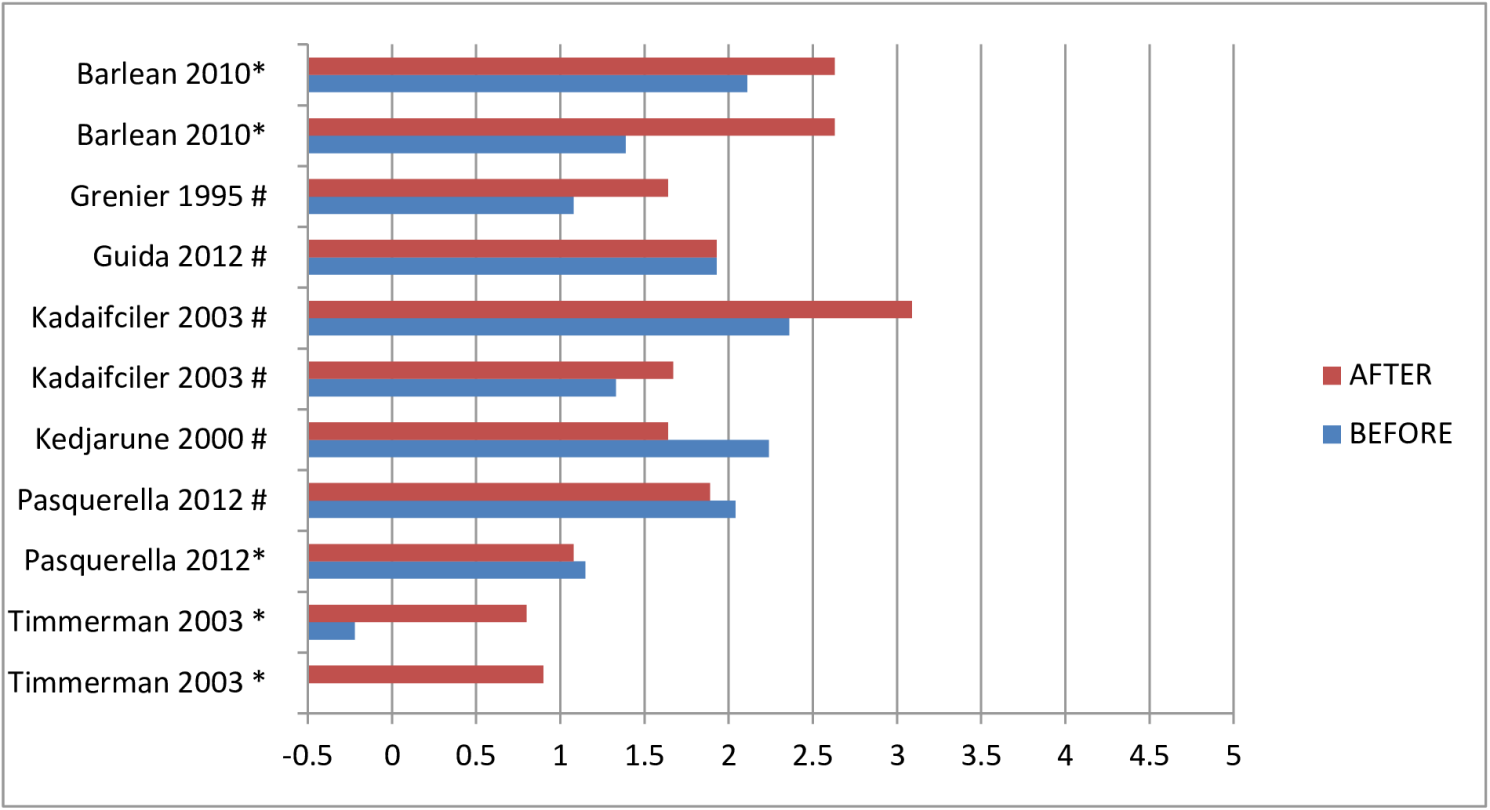
Bacterial or fungal loads in mean Log-10 CFU/m^3^ in dental, measured twice. * = passive sampling method; # active sampling method.

The mean bacterial load in the bio-aerosols ranged from Log 1–3.9 CFU/m^3^ (see Fig 4). Furthermore, six studies analyzed the bio-aerosol contamination before and after treatment. The bacterial or fungal load ranging from Log -0.7–2.4 CFU/m^3^ at baseline and from Log 1–3.1 CFU/m^3^ after treatment (see Fig 5) [25, 68, 71, 72]. Only one study reported on the relation between the distance from the bio-aerosol generating source and the bacterial load. They found a higher bacterial load in the bio-aerosols at 1.5 meter from the oral cavity of the patient than in the bio-aerosols within 1 meter from the patient [26]. One study screened for *B*. *cepacia* and one screened for *M*. *tuberculosis*, however both studies could not retrieve these organisms after regular patient treatment [24, 28].

### Hazard of a bio-aerosol

Seven studies reported on the hazard of micro-organisms to HCWs and/or patients, see Table 5 study characteristics hazard in healthcare and the dental setting [12, 31, 34, 45, 77–79] Three studies looked into the risks for patients when exposed to *Legionella pneumophila* containing sources that may produce bio-aerosols [34, 38, 78]. They reported that cooling towers, air conditioning or mechanical ventilation systems could be a source of *L*. *pneumophila*. Kool *et al*. concluded that patients had an increased risk to acquire *L*. *pneumophila* in hospitals when they used corticosteroids (OR = 13; 95CI% 1.6–102) and when intubated (OR = 10; 95%CI 1.3–73) [34]. Another study identified smoking, drinking alcohol, having chronic lung disease and cancer as risk factors for getting an infection with *L*. *pneumophila* [78]. For the dental clinic there is one case study reported that reported of irreversible septic shock and died after two days in a patient that was infected with *L*. *pneumophila* [79].

**Table 5:** Study characteristics hazard in healthcare and the dental setting.

Study	Set up	Findings
**Augustowska 2006**	Study the variability of airborne microflora of a hospital ward in pneumonological department.Organism:	Decrease of lung function:
	Mesophilic bacteria; fungi	- Decrease spirographic indices in asthmatic patients at increase of bacteria/fungi in air: 37.5%
	Study design:	- Decrease of vital capacity.
	Cross-sectional study with microbiological examination of the air and the lung function in asthmatic patients.	- Decrease of forced expiratory volume in 1 second.
**Browning 2012**	Awareness of the risks involved in treating active herpes labialis in a dental clinic	Case 4:Dental hygienist got both eyes infected with HSV-1. Three possibilities: 1) high exposure to active herpes patients; 2) ultrasonic scaling producing HSV-1 bio-aerosol; 3) rubbing eyes while working.
	Organism: HSV-1	
	Study design: Case series, N = 4	
**Kool 1998**	Investigate a clustered of cases of legionnaires disease among patients at a hospital.	Exposure factors OR univariate:
	Organism: L. *pneumophila*	- Corticosteroids: **5.1 (1–50) SIG**—Intubation: **4.6 (1.1–27) SIG**
	Study design: Case-control study N = 74	- Showering: 0.1 (0–1.3) NS—Medication nebulizer: 1.5 (0.3–6.8) NS
		- Drinking tap water: 1.3 (0.3–5.4) NS
		Exposure factors OR multivariate:
		- Walking in hallway 1–3 days: 0.07 (0.01–0.6) NS- Corticosteroids: **13 (1.6–102) SIG**.
		- Walking in hallway >3 days: 0.3 (0.03–2.6) NS—Intubation: **10 (1.3–73) SIG.**
**MacIntyre 2014**	Description of the range of exposure to HRP in HCWs.	RR infection high risk procedure performed vs not performed:
	Organism: Adenovirus; human metapneumovirus; corona virus; parainfluenza virus; influenza virus A, B; rhinovirus A,B; streptococcus pneumonia; mycoplasma pneumonia; B. pertussis; Legionella spp.; chlamydophilia; Haemophilus influenza type B.	Clinical respiratory infection: **2.5 (1.3–6.5) P<0.01**
		Confirmed virus: 2.8 (0.9–8.7) P = 0.07
	Study design:	Virus or bacteria: **2.6 (1.4–5) P<0.01**
	Prospective study, participants recording following procedures on a daily basis: Provision of nebulizer medications; suction; intubation; bio-aerosol-generating procedures; chest physiotherapy. N = 481	Influenza: 1.5 (0.2–13) NS
		RR bacteria of virus in HCWs:
		Influenza vaccine: 1.3 (0.56–2.8) NS
		Hand washing: 0.7 (0.3–1.63) NS
		HRP performed: **2.9 (1.37–6.22) P = 0.01**
**Palusinska-Szysz 2009**	Description of the pathogenicity of legionella	Symptoms:
	Organism: L. *pneumophila*	*Early*: mild cold; low fever; malaise; anorexia; muscles aches; puss forming sputum; blood streaked sputum; cough blood.
		*Later*: high fever; bronchiolitis; alveolitis; lung damage with infiltrated regions.
	Study design: Review	Risk factors instruments:
		Air conditioning; cooling towers; whirlpool spas; water delivery systems; contaminated birth bathtub; intubation; mechanical ventilation; aspiration; respiratory equipment.
		Risk factors in human:
		neonates; elderly; diabetes; chronic lung disease; chronic severe renal failure; hematologic malignancies; lung cancer; male gender; alcohol; gluco-corticosteroids; immunosuppression; high iron; smokers.
**Ricci, 2012**	Description infection source of legionella	An 82-year-old woman admitted to intensive care unit with fever and respiratory distress. The woman was positive for L. *pneumophila*. The woman has acquired the infection from the contaminated DUWL biofilm and died 2 days after diagnosis.
	Organism: L. *pneumophila*	OR per procedure:
		- Tracheal intubation: 6.6 (2.3–18.9)–SIG
	Study design: Case control	- Non-invasive ventilation: 3.1 (1.4–6.8) SIG
		- Suction before intubation: 3.5 (0.5–24.6) NS
**Tran 2012**	Risk of transmission of acute respiratory infections to HCWs exposed to bio-aerosol generating procedures	- Suction after intubation: 1.3 (0.5–3.4) NS
	Organism: SARS	- Nebulizer: 0.9 (0.1–13.6) NS
	Study design: Systematic review and meta-analysis	- Manipulation oxygen mask: 4.6 (0.6–32.5) NS
		- Bronchoscopy: 1.9 (0.2–14.2) NS
		- Insertion of nasogastric tube: 1.2 (0.4–4.0) NS
		- Chest compression: 1.4 (0.2–11.2) NS
		- Defibrillation: 2.5 (0.1–43.9) NS
		- Chest physiotherapy: 0.8 (0.2–3.2) NS

Abbreviation: HCWs = healthcare workers; HSV-1: herpes simplex virus-1; HRP = high risk procedures; NS = not significant; OR = odds ratio; RR = risk ratio; SARS = severe acute respiratory syndrome; SIG = significant; N = number of subjects.

One systematic review reported on the pooled odds ratio (OR) for the transmission and exposure to Severe Acute Respiratory Syndrome (SARS) in HCWs during bio-aerosol generating procedures. Tracheal intubation (OR = 6.6; 95%CI 2.3–18.9) and noninvasive ventilation (OR = 3.1; 95%CI 1.4–6.8) were risk factors for acquiring SARS. Other bio-aerosol generating interventions such as manipulation of an oxygen mask were not significant risk factors [31]. Another study calculated that the risk ratio for acquiring clinical respiratory infections was 2.5 (95%CI 1.3–6.5) for HCWs performing a high risk procedure [12]. Augustowska *et al*. studied the effect of bacteria and fungi on asthmatic patients. They reported a decrease in maximum breathing capacity due to the increase of bacterial or fungal load in the air [45].

A case-study in a dental clinic described the risk of acquiring Herpes Simplex Virus (HSV)-1 for the dentist and the dental hygienists when they treated a patient with active HSV-1. One member of the treatment team became infected with HSV-1, probably by the bio-aerosol containing HSV-1, induced by ultrasonic scaling or by rubbing her eyes while working. The infected HCWs manifested recurrent HSV-1 infections [77].

## Discussion

By conducting a scoping review we were able to summarize existing evidence on the generation, composition, load and hazards of bio-aerosols in hospital and dental environment. We found that bio-aerosols are generated via multiple sources such as machines, different types of interventions; instruments; and human activity. The composition of bio-aerosols depended on the method of sampling (active versus passive), microbiological techniques (culture based versus DNA-based, different culture plates used) and the setting of the study (specific clinics versus general dental clinics). Bio-aerosols can be hazardous to both patients and HCWs. Multiple studies described the threat of *Legionella* species to elderly and patients with respiratory complaints.

The composition of bio-aerosols was extensively studied in hospital environments (N = 31) compared to dental environments (N = 16). Regarding the micro-organism composition of bio-aerosols, we conclude that bio-aerosols contain a high variety of bacterial and fungal strains from different sources such as the human skin and intestine; and the environment such as soil and water. Based on the sampling and culturing techniques, fungi and Gram-positive bacteria were found most often. Pathogens such as *Legionella* and *Pseudomonas* species were found in bio-aerosols that were distributed by instruments using tap water. Few studies looked for viruses, and in total only ten different viruses were identified, because no open ended detection or identification methods are available for viruses. Therefore only specific targeted techniques were used. Moreover, none of the studies conducted in dental practice have used methods to identify the presence of viruses in the generated aerosols. Therefore, we must keep in mind that the yielded results were dependent on the methodology of the individual study. The results of the individual studies, and the heterogeneity we found in this review, are dependent on the methods leading to an over- or under estimation of the complete bio-aerosol profile. The same inconsistency is discussed in previous studies in which the researchers compared two main sampling methods [80]. The methodological variety between studies, e.g. differences in method of sampling and culturing or sequencing, differences in sampling time and sampling area; and differences in distance to the bio-aerosol generating source caused difficulties in comparing results. When a study used selective medium or agar it results in an overview of selected micro-organisms. This leaves out other micro-organisms that were present at that moment. The same accounts for duration of sampling or passive versus active sampling. In passive sampling, the researcher waits for a certain amount of time for micro-organisms to fall on a Petri dish, while other micro-organisms were still floating in the air and take more time to fall on surfaces. The spread in a bio-aerosol is heterogeneous, so whatever is ‘catched’ on that moment may vary from the second, third or even fourth sampling attempt. So, the method chosen (active or passive) should be dependent on the aim of the air quantification [80]. Furthermore, in many studies variables in the experimental setup were not described, like sampling time, distance and sampling location. Also, no standard deviations of the microbiological loads were reported consistently. Besides, the data might be an underestimation of reality since studies looked for specific micro-organisms, in specific settings by selective sampling and culture dependent techniques, thereby missing other micro-organisms present in the bio-aerosols. Also, there was very little data available on the persistence of micro-organisms in the air over time and the spatial distribution. None of the included studies looked for parasites, although it has been reported that these are present in many tap water dependent bio-aerosol producing systems with plastic tubing [81, 82].

We found little evidence to state the presence or absence of direct threats or health risks for patients or HCWs with regards to bio-aerosols. In the hospital setting, two studies reported on the hazard for the staff [12, 31], and four on the hazard for patients [34, 45, 78, 79]. The search yielded one study for this objective assessing the hazard of an infectious disease to dental staff [77]. However, it is known that on average, dental practitioners carry elevated levels of *Legionella* antibodies [83], but the hazard to non-healthy HCWs and patients remains, based on our findings, unknown. An estimation of the hazard of bio-aerosols is usually made based on the microbial content and load of the bio-aerosols. We conclude that bio-aerosols can be hazardous to certain populations that are extensively exposed to bio-aerosol generating procedures or immunocompromised people.

### Limitations

The search yielded 40 references that were to be screen based on full text. However, we could not recover these 40 full text manuscripts to assess the their eligibility for inclusion, even though we tried to contact the first and/or corresponding author, by retrieving his/her email via the abstract or Google. We assume that the body of evidence for each objective would have been larger if all 40 studies, or at least a part, would have been available and included. Another limitation was that the outcomes and methods were inconsistent throughout all included studies. Also, there was little data on the hazard of bio-aerosols, thus no strong conclusions could be drawn.

### Recommendation for future research

We recommend that future research on bio-aerosols should create an extensive and complete methodology for the quantification of air contamination. Time and frequency of air sampling, distance from sources, location of sampling in both passive and active air sampling techniques should be described thoroughly. Furthermore, the identification of micro-organisms should be done by both selective and non-selective methods and cover organisms that find their origin in water, human, and environment. Also, we believe that infections due to a bio-aerosol should be structurally reported so that the risk for HCWs and patient can be analyzed. Finally, the risks for HCWs, especially dentists, working in an environment in which they are continuously exposed to bio-aerosols, and to their patients remain unclear and therefore need further research. This is needed in order to comprehend the risks of bio-aerosols generated in clinical settings to attention to staff and patients to improve awareness, hygienic standards, risks, and prevention methods.

## Conclusion

Bio-aerosols are generated via multiple sources such as different interventions, instruments and human activity. Bio-aerosols have different microbiological profiles depending on the setting and the used methodology. Bio-aerosols can be hazardous to both patients and healthcare workers. *Legionella* species were found to be a bio-aerosol dependent hazard to elderly and patients with respiratory complaints.

## Supporting information

S1 PRISMA checklist (DOC)
